# Continuous cash dividends, ownership structure and firm value: Evidence from Chinese A-share market

**DOI:** 10.1371/journal.pone.0265177

**Published:** 2022-03-17

**Authors:** Qin Qi, Weijie Li, Chong Liu, Yuncheng Huang, Changsheng Hu

**Affiliations:** 1 School of Economics and Management, Tongji University, Shanghai, China; 2 Institute of Biothermal Science and Technology, University of Shanghai for Science and Technology, Shanghai, China; 3 College of Sciences, Northeastern University, Shenyang, China; 4 CICC Fund Management Co., Ltd., Beijing, China; Institute for Economic Forecasting, Romanian Academy, ROMANIA

## Abstract

This paper examines the relation between continuous cash dividends, ownership structure and firm value across a sample of 1503 firms listed on Chinese A-share market from 2009 to 2017. The empirical results reveal (1) the positive effect of continuous cash dividends on firm value and that (2) values of both state-owned enterprises controlled by central government (SOECGs) and state-owned enterprises controlled by local governments (SOELGs) that distribute continuous cash dividends increase more with ownership concentration than values of those that distribute discontinuous cash dividends; continuous cash dividends fail to mediate the effect of ownership concentration on firm value in private firms (PFs). The results are robust.

## 1 Introduction

Ever since Miller and Modigliani [1] propose dividend irrelevance theory that infers the value of the firm remains unaffected by the dividend policy of the firm, the role of dividend policy in firm valuation has been extensively explored in a large number of studies and the issue remains a hot topic today. While most of the existing literature address either the relation between dividend payout and firm value or the market reaction to the dividend policy or the information content of dividend announcement, how continuous cash dividends affect firm value is rarely studied.

Notably, an increasing number of literature has focused on the relation between ownership structure and firm value and most previous studies are concerned with the monitoring and control mechanism of ownership structure to increase firm value. Until recently, a few papers have addressed the relation between dividend policy, ownership structure and firm value. Sulong and Nor [2] find that in Malaysian market board independence and board size can enhance the monitoring role of dividends, government and foreign ownership in reducing agency costs, thus increasing firm value. Yulianto et al. [3] argue that corporate governance and dividend policy can mitigate the agency problems resulting from ownership structure, thereby increasing firm value. Zulfikar et al. [4] believe that the concentration of ownership weakened the relationship between dividend policy and firm value in Indonesia market. However, much less is known about how continuous cash dividends affect the relation between ownership structure and firm value in China.

For the following two reasons, China is a suitable area to explore the effect of continuous cash dividends on firm value and on the relation between ownership concentration and firm value.

First, firms listed on Chinese A-share market, one of the leading emerging markets in the world, have poor tradition of dividend payments. Their cash dividend payments are discontinuous and unstable. The China Securities Regulatory Commission (CSRC) hence issued a series of regulations on cash dividends in 2004, 2006 and 2008 respectively to improve the situation and gave verbal warnings or punished those listed companies which violate the regulations up till now. Actions to regulate listed companies’ cash dividend payments are still ongoing, and this research can help firm managers, investors and researchers understand the importance of continuous cash dividends.

Second, in a survey of corporate governance in China, Jiang and Kim [5] document that the dominant agency problem in China is the horizontal agency conflict between controlling shareholders and minority shareholders due to concentrated ownership structure and poor law and regulations in Chinese stock market. Wei et al. [6] find that agency problems in China are more serious as the separation of control rights and cash flow rights by insiders is relatively larger and legal protection of outside shareholders is relatively weaker. Researches related to ownership structure demonstrate that the solution to agency problems differs significantly among different types of controlling shareholders, hereby affecting firm performance differently [7]. In addition, existing empirical studies show that the relation between ownership concentration and firm value is subject to the types of controlling shareholders [8]. Jiang and Kim [5] believe that minority shareholders in China must rely on strong laws and regulations to protect their interest as boards, institutional investors, banks, security analysts, independent auditors and the media, which have explicit or implicit political connections with the state, play a limited role in protecting minority shareholders’ interest. Continuous cash dividends as one way of legal protection of minority shareholders and one part of stable dividend policy can help reduce agency costs and improve corporate governance, thus increasing firm value.

In this paper, we use a large sample of 13527 firm years of firms listed on Chinese A-share market including both the Shanghai stock exchange and Shenzhen stock exchange to investigate the effect of continuous cash dividends on firm value with the pooled sample and the effect of continuous cash dividends on the relation between ownership concentration and firm value with three subsamples. The empirical results show continuous cash dividends are positively related to firm value, values of both SOECGs and SOELGs that distribute continuous cash dividends increase more with ownership concentration than values of those that distribute discontinuous cash dividends and that continuous cash dividends fail to mediate the effect of ownership concentration on firm value in PFs.

The study contributes to the existing literature in the following aspects. First, this paper adds to literature the understanding of how continuity of cash dividends affects firm value and how continuous cash dividends affect the relationship between ownership concentration and firm value differently based on different ownership types of firms listed on Chinese A-share market. Second, it is not enough to study how the relation between ownership concentration and firm value is affected by continuous cash dividends in state-owned enterprises (SOEs) and non-SOEs. So the paper extends SOEs (equivalent to government-controlled firms) to SOECGs and SOELGs according to the distinct features of state assets in China, and the latter is rarely studied in the discussion of the relation between dividend policy, ownership structure and firm value. Third, it has potential policy implications to explore the effect of continuous cash dividends on firm value and on the relation between ownership concentration and firm value.

The remainder of the paper proceeds as follows. Section 2 presents a brief introduction to institutional background and articulates the hypotheses. Section 3 describes data and summary statistics of relevant variables. Section 4 demonstrates research methodology, the empirical results and then reports the robustness test results. Section 5 concludes the paper.

## 2 Institutional background and Hypothesis development

### 2.1 The CSRC’s regulations on cash dividends

In 2004, the CSRC stated that company issuing new shares or convertible bonds will not be approved if cash or stock dividends were not paid in the past three years. The CSRC went further in 2006, stating that the issuance of new shares by listed firms shall be consistent with the provision that the firms’ accumulatively distributed cash or stock dividends over the past three years must be higher than 20% of the average realized annual distributable profits.

In 2008, the CSRC introduced a significant regulatory change restricting to distribution of cash dividends that if firms seek to raise new equity capital, their accumulatively distributed cash dividends in the past three years must be higher than 30% of the average realized annual distributable profits. In addition, cash dividend policy must be clearly written in Articles of Incorporation and cash dividend payments should be continuous and stable.

### 2.2 Ownership structure in China

Chinese A-share market refers to Shanghai Stock Exchange founded in 1990 and Shenzhen Stock Exchange founded in 1991. In the beginning all companies listed on the two stock exchanges were SOEs with a split-share structure of non-tradable shares (NTS) and tradable shares (TS). State shares, legal person shares and employee shares are non-tradable shares and public A-shares, B-shares, H-shares and other foreign shares are tradable shares [9]. In order to reduce the controlling shareholders’ expropriation of minority shareholders’ interest, the split-share structure reform took place in 2005, which began to convert NTS into TS. As China’s economy grows rapidly, more and more PFs are listed on the two stock exchanges mentioned above.

The main ownership types of listed firms on Chinese A-share market include SOECGs, SOELGs and PFs. SOECGs are on a higher administrative level in comparison with SOELGs. In 1995, the central government followed the policy of “retaining the large, releasing the small”, which meant the state just kept a few hundred largest SOEs in the strategic industries (including public utilities, transportation, energy and heavy industry etc.) and handed over other SOEs in less strategic industries to the local governments. In contrast, PFs originate mainly from family-owned firms in the non-strategic industries. Besides, they have different economic emphases. SOECGs keep a closer eye on the political goals and the overall economic development as theoretically speaking shares of SOECGs are owned by all residents of China and the state just acts an agent to protect all the Chinese people’s interest [6]. SOELGs focus more on the local economy and the local people’s welfare, providing part of fiscal revenue for the local governments. The ultimate goal of PFs is to maximize all the shareholders’ interest.

In comparison with other stock markets in developed countries like the United States or England, Chinese listed firms have fewer foreign and institutional investors. Foreign shares are shares owned by non-mainland Chinese investors, including foreign investors and investors with Hongkong, Macau and Taiwan residency. It is believed that foreign investors bring in knowledge and experience and improve corporate governance. Therefore they are deemed to be value increasing. Institutional shares are shares owned by Chinese domestic legal entities, including domestic mutual funds, insurance companies, government agencies and other enterprises. Many of these legal entities are fully or partially owned by different levels of government (provincial, municipal or county). In the meantime, institutional shareholders are more profit-oriented and may have more incentives to monitor the firms. So how institutional shares affect firm value requires an empirical answer [6].

### 2.3 Hypothesis development

A group of researchers argue that cash dividend is positively related to firm value. As Baker and Wurgler [10], Shefrin et al. [11] suggest that stock prices positively correlate with cash dividend payouts. Yilmaz and Gulay [12] find positive price reactions before the cash dividend payment date. Al-Yahyaee et al. [13] document a significant positive share price reaction to the announcement of increase of cash dividends. Xiong [14] believes that the increase of cash dividend payout positively affects the relation between institutional investor and firm value. Luo [15] proves that institutional investors’ shareholding is positively related to the company’s market value on the condition that a listed company can pay cash dividends continuously.

In addition, Chinese evidence shows that continuous cash dividends play an important role in firm valuation. First, continuous cash dividends can improve the relevance of the stock return volatility to the fundamental variables [16]. Second, continuous payments of cash dividend bring about positive effect on the investments so as to promote firm value [17]. Third, continuous payments of cash dividend by listed firms are good for the promotion of stocks’ pricing efficiency and significant in influencing investors’ behaviors and market value management [18]. The preceding analysis yields the first hypothesis to test in this paper.

Hypothesis 1: Firm value positively correlates with continuous cash dividends.

In classic literature, agency theory is central to the analysis of the relation between ownership structure and firm value. As La Porta, Lopez-de-Silanes Schleifer and Vishny [19] report, the agency conflicts between controlling shareholders and minority shareholders are omnipresent and more severe in many countries outside U.S due to lack of strong legal protections and other governance mechanisms, which make cash holding a key factor for the financial stability of companies. The excess cash holdings enable the managements to overinvest, pursue their private benefits and harm shareholders’ interest, aggravating the agency problems in the companies. When the agency cost is relatively higher, the resources in the company are less likely to be used to maximize shareholders’ value and excess cash holdings are negatively associated with firm performance. In contrast, when the agency cost is relatively lower, excess cash won’t be misused and excess cash holdings can improve firm performance. La Porta, Lopez-de-Silanes, Schleifer and Vishny (LLSV) [20] use the effect of law on controlling shareholders’ expropriation of minority shareholders to explain dividend. As legal protection of minority shareholders pushes controlling shareholder to pay minority shareholders cash dividends. In addition, controlling shareholder are willing to pay cash dividends to show their concern over minority shareholders with the purpose of attracting investors when issuing new shares. Easterbrook [21] believes firm value can be enhanced by reducing agency costs when part of excess cash is used to pay cash dividends, pushing the company to finance in the capital market and transform the company’s capital structure in order to make debtors supervise the companies and new investors believe the managements are capable of allocating the capital efficiently. Easterbrook argues that dividends can be either the result or the solution to agency conflicts. Dividend payout can contribute to mitigate agency conflicts. Jensen [22] documents that higher dividends provide a cost-effective substitute to shareholder monitoring.

From the perspective of agency costs, PFs possess the least agency costs, followed by SOELGs and SOECGs in China. As SOECGs are under extremely strict and discreet supervision from central government and their operations are often interfered by central government and SOELGs are under relatively looser supervision for the local governments depend more on SOELGs to develop local economy, which is a key index measuring local government officials’ performance and achievements and a determining factor for their political promotions, the agency costs of SOECGs are higher than those of SOELGs. Meanwhile, PFs possess more independent board and balanced shareholdings among blockholders as well as clearer property rights [23], so the agency costs of PFs are the least.

Chinese evidence proves the arguments that shares of SOECGs are significantly negatively related to firm value and shares of PFs are significantly positively associated with firm value. In reality, SOELGs are subject to more government interventions than PFs and have less agent costs than SOECGs. The effect of ownership structure on firm value in SOELGs remains an empirical question. The preceding analysis in addition to Hypothesis 1 yields Hypothesis 2a, Hypothesis 2b, and Hypothesis 2c to test in this paper.

Hypothesis 2a: Values of SOECGs with continuous cash dividends decrease less with ownership concentration than values of those without continuous cash dividends.

Hypothesis 2b: Values of SOELGs with continuous cash dividends increase more with ownership concentration than values of those without continuous cash dividends.

Hypothesis 2c: Values of PFs with continuous cash dividends increase more with ownership concentration than values of those without continuous cash dividends.

## 3 Data and summary statistics

The data for this study are retrieved from the China Center for Economic Research (CCER) database and Choice database, commercially available at Eastmoney Information Co.,Ltd. in China. The initial sample consists of all A-share common stocks publicly traded on the Shanghai Stock Exchange and Shenzhen Stock Exchange for the period 2009–2017. We then adjust the sample by excluding financial firms, ST listed firms, firms cross-listed on both Chinese A-share and B-share markets or cross-listed on both Chinese A-share market and Hong Kong stock market. In the meantime, the sample also meets the following criteria: (1) The firms are required to be listed on the Chinese A-share market for more than 5 years (listed in 2012 and before). (2) The annual trading time of the firms should be at least 40 weeks. (3) The net annual value of the firms should be positive. The final sample includes 13527 firm-year observations representing 1503 unique firms.

Panel A of Table 1 reports summary statistics of the main variables in the analyses, including median and mean values. The mean market to book ratio (MB) of the sample is 0.504 (median, 0.487). The mean value of continuous cash dividends (DIV_5) is 0.604, indicating that 60.4% firms in the sample distribute cash dividends for 5 straight years on average. The mean value of shareholding ratio of the largest shareholder (OWN1) is 35.3% (median, 33.7%). Panel A of Table 1 also presents the mean and median values of control rights (CONTROL), cash flow rights (CASH), foreign ownership (FOREIGN) and institutional ownership (INSTITUTIONAL) as well as tradable share ratio (TSH), firm size (SIZE) and board duality (DUALITY). The mean control rights of the sample is 39.6% (median, 38.7%). The mean cash flow rights of the sample is 33.4% (median, 32.8%). The mean foreign ownership of the sample is 2.7% (median, 0%). The mean institutional ownership of the sample is 29.8% (median, 26.8%).

**Table 1:** Summary statistics.

**Panel A: firm characteristics**						
**Variable**	**No of Obs.**	**Median**	**Mean**	**SD**	**Min**	**Max**
**MB**	13,527	0.487	0.504	0.278	0	1.846
**DIV_5**	13,527	1	0.604	0.489	0	1
**OWN1**	13,527	0.337	0.353	0.175	0.013	0.899
**TSH**	13,527	0.810	0.738	0.326	0.037	1
**SIZE**	13,527	0.015	0.015	0.656	0.271	3.859
**DUALITY**	13,527	0	0.242	0.428	0	1
**CONTROL**	13,527	0.387	0.396	0.188	0.063	1
**CASH**	13,527	0.328	0.334	0.189	0	1
**FOREIGN**	13,527	0	0.027	0.096	0	0.890
**INSTITUTIONAL**	13,527	0.268	0.298	0.246	0	0.926

Panel B: comparison of firm value	MB	
	Median	Mean
**Firms with continuous cash dividends**	0.493	0.534
**Firms with discontinuous cash dividends**	0.477	0.503
**Difference**	1.440*	6.275***
**SOECGs**	0.494	0.513
**SOELGs**	0.394	0.417
**Difference**	12.826***	11.605***
**PFs**	0.528	0.542
**SOECGs**	0.494	0.513
**Difference**	4.369***	17.621***
**PFs**	0.528	0.542
**SOELGs**	0.394	0.417
**Difference**	23.902***	20.826***

Note that

*, **, ***denote significance at the 10%, 5% and 1% level respectively. N = 13,527.

Panel B of the Table 1 presents the first test of effect of continuous cash dividends or ownership concentration on firm value by comparing firm value among two cash dividend payment groups and three different ownership type groups. The table also presents *t*-statistics and Wilcoxon *z*-statistics for difference in median and mean tests respectively.

**Fig 1:**
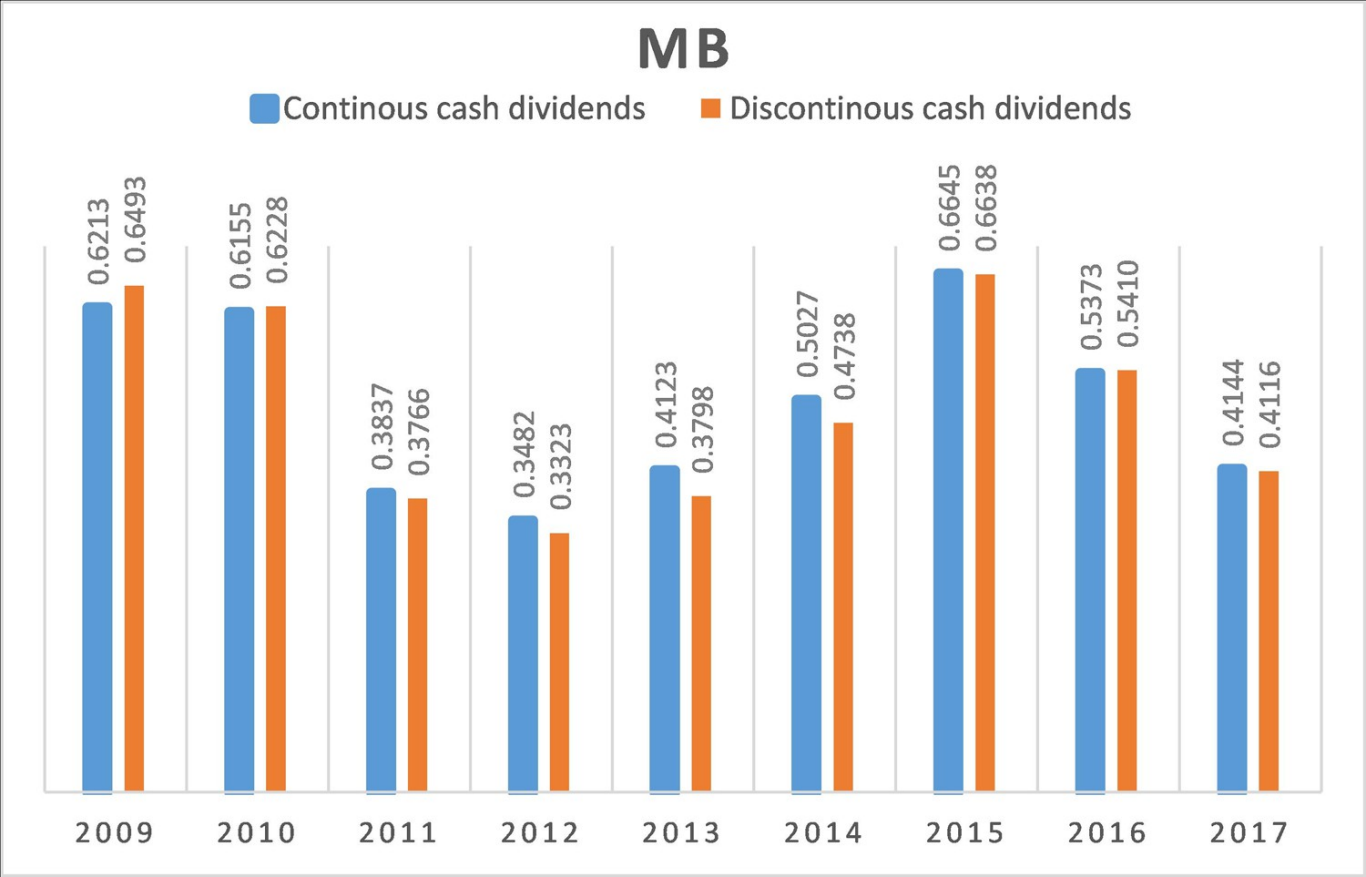
Firm value from 2009 to 2017.

Firms with continuous cash dividends have a median MB of 0.493, which is significantly larger than the median MB of 0.477 of firms with discontinuous cash dividends. This is also true for mean measures. Besides, this relation appears to hold over time. Fig 1 shows the median MB of firms with continuous cash dividends and discontinuous cash dividends from 2009 to 2017. It can be seen that firms with continuous cash dividends have a higher firm value than those with discontinuous cash dividends for six out of nine years.

The median firm value of PFs is the highest, followed by SOECGs, and lastly SOELGs in terms of MB. Specifically, the median 0.528 MB of PFs is significantly higher than the median 0.494 MB of SOECGs and the median 0.394 MB of SOELGs. The results are similar for mean measures.

In summary, the values of sample firms show that firms with continuous cash dividends have a significantly higher MB than those with discontinuous cash dividends; PFs possess the highest firm value, followed by SOECGs, and lastly SOELGs.

Table 2 presents the Pearson correlation coefficients between the dependent variable and the key testing variables. It is shown that OWN1, TSH and CONTROL are highly correlated to MB, since they are important factors affecting firm value. The Pearson correlation coefficient between DIV_5 and MB is -0.008 and insignificant. And the remaining key testing variables are significantly and positively related to MB. So we find evidence that ownership concentration is positively associated with firm value and need to find further evidence to see how continuous cash dividends is associated with firm value with addition of controls.

**Table 2:** Correlation analysis.

	MB	DIV_5	OWN1	TSH	SIZE	DUALITY	CON-TROL	CASH	FOREIGN	INSTITU-TIONAL
**MB**	1									
**DIV_5**	-0.008 (0.39)	1								
**OWN1**	0.225*** (0.00)	0.018** (0.04)	1							
**TSH**	0.246*** (0.00)	-0.025*** (0.00)	0.278*** (0.00)	1						
**SIZE**	0.085*** (0.00)	0.037*** (0.00)	0.476*** (0.00)	0.578*** (0.00)	1					
**DUALITY**	0.061*** (0.00)	-0.012 (0.17)	-0.099*** (0.00)	-0.138*** (0.00)	-0.164*** (0.00)	1				
**CONTROL**	0.216*** (0.00)	0.017* (0.04)	0.724*** (0.00)	0.166*** (0.00)	0.364*** (0.00)	-0.069*** (0.00)	1			
**CASH**	0.196*** (0.00)	0.034*** (0.00)	0.622*** (0.00)	0.084*** (0.00)	0.317*** (0.00)	-0.073*** (0.00)	0.824*** (0.00)	1		
**FOREIGN**	0.050*** (0.00)	0.035*** (0.00)	0.124*** (0.00)	0.019** (0.03)	0.054*** (0.00)	-0.020** (0.02)	0.154*** (0.00)	0.091*** (0.00)	1	
**INSTITU-TIONAL**	0.137*** (0.00)	0.059*** (0.00)	0.391*** (0.00)	0.745*** (0.00)	0.507*** (0.00)	-0.169*** (0.00)	0.279*** (0.00)	0.178*** (0.00)	0.123*** (0.00)	1

The p-values are reported in parenthesis, where

*, ** and *** represent significance at the 10%, 5%, and 1% level respectively.

## 4 Methodology and empirical results

### 4.1 Methodology

Eq (1) is to examine the effect of continuous cash dividends on firm value with the pooled sample. It is theoretically and empirically suggested that there is a nonlinear relation between ownership and firm value [24–27]. The addition of the square value of OWN1 in the Eq (1) is to test whether there exists a non-linear relation between ownership concentration and firm value. Eq (2) is to explore the effect of continuous cash dividends on the relation between ownership concentration and firm value with three subsamples (including SOECGs, SOELGs and PFs).


$${FV}_{i,t}={\alpha_{0}+\alpha_{1}DIV\_5}_{i,t}+{\alpha_{2}OWN1}_{i,t}+\alpha_{3}{OWN1}_{i,t}^{2}+\Sigma \lambda {Controls}_{i,t}{+\varepsilon }_{i,t}$$ (1)

$${FV}_{i,t}=\alpha_{0}+{\alpha_{1}DIV\_5}_{i,t}+{\alpha_{2}{OWN1}_{i,t}*DIV\_5}_{i,t}+{\alpha_{3}OWN1}_{i,t}+\alpha_{4}{OWN1}_{i,t}^{2}+\Sigma \lambda {Controls}_{i,t}+\varepsilon_{i,t}$$ (2)

where i indexes firm (1,2,…1503) and t indexes year (2009, 2010, …2017).

We measure firm value with market to book ratio (MB) as Fama and French [28], Baker and Wurgler [29] and Pinkowitz, et al. [30] do in their research, which is computed as logarithm of the sum of market value equity and total debt, all divided by total asset.

Following previous studies [16,18], we define continuous cash dividends (DIV_5) as a dummy variable and take the value of one if the firm distributes cash dividend for 5 straight years from 2013 to 2017 and zero if firms distribute cash dividends for one or two fiscal years.

Following Woidkte [31] and Luo [32], we measure ownership concentration (OWN1) with the largest shareholder’s holdings divided by total shares.

In addition to continuous cash dividends, ownership structure and firm value variables, we use other firm-specific characteristics that might explain firm value as controls. Tradable share ratio (TSH) is tradable shares divided by total shares. Firm size (SIZE) is log of total asset. Board duality (DUALITY): we capture this effect by a dummy variable and take the value of one if board chairman is also CEO of the firm, and zero otherwise. Control rights (CONTROL) are the sum of the minimum of voting rights on each control chain. Cash flow rights (CASH) are the sum of a controlling owner’s percentage of ownership over profits, losses and dividends of the firm from each control chain. Foreign ownership (FOREIGN) is shares held by non-mainland Chinese investors divided by total shares. Institutional ownership (INSTITUTIONAL) is shares held by Chinese domestic legal entities divided by total shares. YEAR: The sample period covers nine years, so we capture this effect by eight dummy variables. INDUSTRY: We capture this effect by sixteen dummy variables according to the Chinese Securities Regulatory Commission industry classification.

### 4.2 Effect of continuous cash dividends on firm value

We regress firm value on the *DIV_5* dummy variable and the control variables. Results are reported in Table 3. Column (1) shows the estimates using MB as the measure of firm value. The *DIV_5* coefficient is positive and significant at 5% level. Therefore, H1 is supported and we believe that continuous cash dividends matter in firm valuation. Column (1) also demonstrates that ownership concentration is positively associated with firm value and the results are significant at 1% level. The coefficient of *OWN1*^*2*^ in column (2) is -2.1621 and significant at 1% level, which indicates a non-linear relation between ownership concentration and firm value. The results document an overall significant inversed U shape relation between ownership concentration and market to book ratio with an average reflection point of 42.90%. The adjusted R^2^ in column (2) shows that the independent variables combined can explain 36.23% of variation in firm value. In the meantime, the *DIV_5* coefficient in column (2) is 0.0093 and significant at 5% level, indicating firm value may increase 0.93% when the firms distribute cash dividends for 5 straight years.

**Table 3:** The relation between continuous cash dividends, ownership concentration and firm value.

	MB		
	(1)	(2)	
**DIV_5**	0.0087** (2.11)	0.0093** (2.37)
**OWN1**	0.3106*** (17.30)	1.8547*** (41.80)
**OWN1** ^**2**^		-2.1621*** (-37.70)
**TSH**	0.3453*** (32.58)	0.1894*** (17.38)
**SIZE**	-0.0643*** (-14.42)	-0.0882*** (-20.58)
**DUALITY**	0.0481*** (10.15)	0.0378*** (8.37)
**CONTROL**	0.1077*** (5.02)	0.0550*** (2.69)
**CASH**	0.1555*** (8.23)	0.1425*** (7.93)
**FOREIGN**	0.1001*** (4.71)	0.0964*** (4.76)
**INSTITUTIONAL**	-0.1813*** (-13.90)	-0.0630*** (-4.92)
**Constant**	0.2164*** (8.83)	0.2042*** (8.76)
**Year**	YES	YES
**Industry**	YES	YES
**No.of Obs.**	13527	13527
**Adj. R** ^**2**^	0.2952	0.3623

T-statistics are in the parenthesis.

*, **, ***denote significance at the 10%, 5% and 1% level respectively.

Note that the coefficients of the control variables generally have the expected signs. Large firms have more agency costs, doing harm to firm value, so firm size may negatively affect firm value. CEO duality is good for firm value as the unity of the dual role of owner and manager may lower agency costs. The higher the cash-flow rights are, the stronger the alignment-of-interest effect is, reducing agency costs and increasing firm value. So cash-flow rights are positively related to firm value. Institutional ownership is significantly negatively related to firm value, which is consistent with the fact that many of the firms’ legal entities are fully or partially owned by different levels of government in China. We also find tradable share ratio, control rights, foreign ownership have a positive impact on firm value.

### 4.3 Effect of continuous cash dividends on the relation between ownership concentration and firm value

To further investigate the effect of continuous cash dividends on the relation between ownership concentration and value of firms of different ownership types, we divide the pooled sample into three subsamples resulting in the subsamples of SOECGs, SOELGs and PFs. We run regressions of firm value on continuous cash dividends, ownership concentration, the interaction between continuous cash dividends and ownership concentration, and control variables. The results are reported in Table 4. The focus is now on the coefficient of the interaction term DIV_5*OWN1. If the coefficient of the interaction term DIV_5*OWN1 is positive and significant, it can be concluded that values of firms with continuous cash dividends increase more with ownership concentration than values of those with discontinuous cash dividends.

**Table 4:** Effect of continuous cash dividends on the relation between ownership concentration and firm value.

	MB	MB		
	(1) Pooled	(2) SOECGs	(3) SOELGs	(4) PFs
**DIV_5**	-0.0087 (-1.06)	-0.0409 (-1.43)	-0.0880*** (-4.46)	0.0062 (0.66)
DIV_5*OWN1	0.0561** (2.50)	0.1791** (2.52)	0.2450*** (5.17)	-0.0145 (-0.51)
**OWN1**	1.8249*** (39.73)	2.0051*** (13.08)	1.0165*** (10.58)	1.8896*** (30.78)
**OWN1** ^**2**^	-2.1662*** (-37.77)	-2.5779*** (-13.30)	-1.4016*** (-12.69)	-2.1503*** (-26.63)
**TSH**	0.1889*** (17.34)	0.0615* (1.79)	-0.0097 (-0.45)	0.2006*** (14.30)
**SIZE**	-0.0884*** (-20.62)	-0.1909*** (-19.46)	-0.1015*** (-13.25)	-0.0529*** (-8.66)
**DUALITY**	0.0375*** (8.31)	-0.0643*** (-3.91)	0.0075 (0.59)	0.0350*** (6.82)
**CONTROL**	0.0538*** (2.63)	-0.2134*** (-2.86)	0.0903 (1.64)	-0.0402 (-1.64)
**CASH**	0.1411*** (7.85)	0.2127*** (4.75)	0.0969** (2.48)	0.2226*** (9.59)
**FOREIGN**	0.0958*** (4.74)	0.0191 (0.21)	0.0379 (0.69)	0.0352 (0.81)
**INSTITUTIONAL**	-0.0635*** (-4.96)	0.1646*** (4.10)	0.1410*** (5.54)	-0.0298 (-1.76)
**Constant**	0.2187*** (9.11)	0.6572*** (8.13)	0.4999*** (12.87)	0.1762*** (5.29)
**Year**	YES	YES	YES	YES
**Industry**	YES	YES	YES	YES
**No.of Obs.**	13527	1728	3105	8073
**Adj. R** ^**2**^	0.3626	0.3878	0.2853	0.4576

T-statistics are in the parenthesis.

*, **, ***denote significance at the 10%, 5% and 1% level respectively.

Table 4 shows that the coefficients of DIV_5*OWN1 in column (2) and column (3) are both positive and significant at 5% level and 1% level respectively, meaning values of SOECGs and SOELGs that distribute continuous cash dividends increase more with ownership concentration than values of those with discontinuous cash dividends. Therefore, H2a is rejected and H2b is supported. Specifically, values of SOECGs with continuous cash dividends increase 17.91% more with ownership concentration than values of those with discontinuous cash dividends. Values of SOELGs with continuous cash dividends increase 24.50% more with ownership concentration than values of those with discontinuous cash dividends. The coefficient of DIV_5*OWN1 in PFs is negative but statistically insignificant, indicating continuous cash dividends fail to mediate the effect of ownership concentration on firm value in PFs. Therefore, H2c is rejected.

### 4.4 Endogeneity of ownership concentration and firm value

The results may be biased if ownership concentration and firm value are endogenously determined. We use *OWN*1_*i,t*−1_ as instrumental variable to repeat the regression of MB on continuous cash dividends, ownership concentration and control variables. The results in Table 5 show that the coefficients of DIV_5*OWN1 in column (2), column (3) are still both positive and significant at 5% level, 1% level respectively and the coefficient of DIV_5*OWN1 in column (4) is positive but insignificant. In general, our results are robust.

**Table 5:** Endogeneity of ownership concentration and firm value: Using OWN_i, t-1_ as instrument variable.

	MB	MB		
	(1) Pooled	(2) SOECGs	(3) SOELGs	(4) PFs
**DIV_5**	-0.0146* (-1.68)	-0.0218 (-0.73)	0.0630*** (-3.16)	-0.0079 (-0.77)
DIV_5*OWN1	0.0678*** (2.86)	0.1288* (1.75)	0.1939*** (4.07)	0.0256 (0.83)
**OWN1**	0.8123*** (18.28)	1.0238*** (6.82)	0.3719*** (4.00)	0.6347*** (10.76)
**OWN1** ^**2**^	-1.1356*** (-19.23)	-1.560*** (-7.78)	-0.7036*** (-6.33)	-0.8225*** (-9.96)
**TSH**	0.1221*** (9.95)	-0.0521 (-1.38)	-0.0964*** (-4.06)	0.1604*** (10.04)
**SIZE**	-0.0915*** (-20.37)	-0.2108*** (-20.51)	-0.1270*** (-16.02)	-0.0372*** (-5.83)
**DUALITY**	0.0432*** (8.91)	-0.0637*** (-3.72)	0.0036 (0.28)	0.0455*** (8.07)
**CONTROL**	0.1477*** (7.06)	-0.1216* (-1.76)	0.1318*** (2.56)	0.0754*** (2.88)
**CASH**	0.1390*** (7.22)	0.2189*** (4.65)	0.0287 (0.72)	0.2609*** (10.23)
**FOREIGN**	0.0841v (3.96)	-0.0091 (-0.10)	0.0183 (0.33)	0.0656 (1.40)
**INSTITUTIONAL**	-0.0039*** (-0.28)	0.2478*** (5.69)	0.1958*** (7.15)	0.1567 (0.85)
**Year**	YES	YES	YES	YES
**Industry**	YES	YES	YES	YES
**Constant**	0.4204 (16.03)	0.9198*** (10.75)	0.6835*** (16.48)	0.3586*** (9.63)
**No.of Obs.**	12024	1536	2760	7176
**Adj.R** ^**2**^	0.2807	0.4023	0.2842	0.3206

Z-statistics are in the parenthesis.

*, **, *** denote significance at the 10%, 5% and 1% level respectively.

### 4.5 Firm-fixed effect test

The findings may be driven by omitted firm-specific variables that correlate ownership concentration and firm value. To alleviate this concern, we run panel regressions of MB on continuous cash dividends, ownership concentration, the interaction between continuous cash dividends and firm value, and control variables with firm-fixed effects.

The results in Table 6 are similar to those in Table 4. The coefficients of DIV_5*OWN1 in column (2) and column (3) are still both positive and significant at 10% level and 1% level respectively. The coefficient of DIV_5*OWN1 in column (4) is positive and statistically insignificant. Robust to firm fixed effects, values of SOECGs and SOELGs that distribute continuous cash dividends increase more with ownership concentration than values of those with discontinuous cash dividends and continuous cash dividends fail to mediate the effect of ownership concentration on firm value in PFs.

**Table 6:** Robustness test: Firm-fixed effect results.

	MB	MB		
	(1) Pooled	(2)SOECGs	(3)SOELGs	(4)PFs
**DIV_5**	-0.0535 (-0.72)	-0.1759** (-2.03)	-0.1750** (-2.28)	-0.0440 (-0.58)
DIV_5*OWN1	0.0941*** (3.70)	0.1620* (1.74)	0.3658*** (5.20)	0.0487 (1.63)
**OWN1**	1.8043*** (33.44)	2.0469*** (10.47)	1.3132*** (10.34)	2.0006*** (28.59)
**OWN1** ^**2**^	-2.0307*** (-28.50)	-2.6304*** (-10.18)	-1.5101*** (-10.85)	-2.2340*** (-22.85)
**TSH**	0.1261*** (11.01)	-0.0420 (-1.06)	0.0124 (0.48)	0.0894*** (6.10)
**SIZE**	0.0722*** (9.99)	-0.0500** (-2.35)	0.0265* (1.72)	0.0524*** (5.61)
**DUALTIY**	0.1293** (1.75)	-0.1217* (-1.72)	0.0480 (0.71)	0.1305* (1.74)
**CONTROL**	0.1133*** (4.61)	0.1006 (1.17)	-0.0179 (-0.25)	0.1073*** (3.59)
**CASH**	0.0930*** (4.50)	0.1533*** (2.71)	0.1936*** (4.20)	0.0949*** (3.63)
**FOREIGN**	-0.0441 (-1.31)	0.3631*** (2.74)	-0.0981 (-0.98)	-0.2003*** (-3.45)
**INSTITUTIONAL**	0.0026 (0.19)	0.2557*** (5.66)	0.0604** (2.12)	0.0515*** (2.87)
**Constant**	0.2473* (1.78)	0.7503*** (5.95)	0.6895*** (7.33)	0.2455* (1.74)
**Firm**	YES	YES	YES	YES
**Year**	YES	YES	YES	YES
**Industry**	YES	YES	YES	YES
**No.of Obs.**	13527	1728	3105	8073
**Adj. R** ^**2**^	0.6731	0.6847	0.6607	0.6941

T-statistics are in the parenthesis.

*, **, *** denote significance at the 10%, 5% and 1% level respectively.

### 4.6 Alternative variable measures

In this section, we check the robustness of the results from the perspective of alternative variable measures. That is, we vary the way of measures of firm value and ownership concentration.

First, we use Tobin’s Q as an alternative proxy for firm value, which is widely used and computed as the sum of tradable shares multiplying year-end closing price of the share, non-tradable shares multiplying book value per share, and book value of total debt, all divided by book value of total assets for it is known that the shares issued by firms listed on Chinese A-share market could roughly be classified into TS and NTS. The results shown in Table 7 are similar to those in Table 4. The coefficients of DIV_5*OWN1 in column (2) and column (3) are still both positive and significant at 1% level. The coefficient of DIV_5*OWN1 in column (4) is positive and statistically insignificant.

**Table 7:** Robustness test: Tobin’s Q as an alternative measure of firm value.

	Tobin’s Q	Tobin’s Q		
	(1) Pooled	(2) SOECGs	(3) SOELGs	(4) PFs
**DIV_5**	-0.0654 (-1.48)	-0.5924*** (-2.87)	-0.1275 (-1.46)	-0.0083 (-0.16)
DIV_5*OWN1	0.3265*** (2.70)	1.4939*** (2.91)	0.6364*** (3.04)	0.0563 (0.36)
**OWN1**	3.9507*** (15.99)	4.548*** (4.12)	1.4561*** (3.42)	4.2857*** (12.84)
**OWN1** ^**2**^	-4.5709*** (-14.82)	-5.7517*** (-4.12)	-1.6250*** (-3.32)	-4.7275*** (-10.77)
**TSH**	2.4121*** (41.17)	2.0216*** (8.16)	1.3549*** (14.00)	2.7162*** (35.60)
**SIZE**	-0.9559*** (-41.46)	-1.2963*** (-18.32)	-0.7215*** (-21.26)	-1.009*** (-30.41)
**DUALITY**	0.1624*** (6.68)	-0.3691*** (-3.11)	0.1317** (2.35)	0.1514*** (5.43)
**CONTROL**	0.1228 (1.12)	-1.5597*** (-2.90)	-0.1393 (-0.57)	-0.0629 (-0.47)
**CASH**	0.4698*** (4.86)	0.9044*** (2.80)	0.4833*** (2.79)	0.6857*** (5.43)
**FOREIGN**	0.1224 (1.13)	0.0916 (0.14)	-0.0063 (-0.03)	-0.4731** (-2.00)
**INSTITUTIONAL**	0.2521*** (3.66)	0.5146* (1.78)	0.7968*** (7.07)	0.4099*** (4.45)
**Constant**	0.3830*** (2.97)	1.9594*** (3.36)	1.3565*** (7.88)	-0.0265 (-0.15)
**Year**	YES	YES	YES	YES
**Industry**	YES	YES	YES	YES
**No.of Obs.**	13527	1728	3105	8073
**Adj. R** ^**2**^	0.3972	0.3391	0.3612	0.4683

T-statistics are in the parenthesis.

*, **, *** denote significance at the 10%, 5% and 1% level respectively.

Second, we use the top ten largest shareholders’ holding ratio (OWN10) as an alternative measure for OWN1 and repeat the original MB analysis of Table 4. The results shown in Table 8 are similar to those in Table 4. The coefficients of DIV_5*OWN10 in column (2) and column (3) are still both positive and significant at 1% level. The coefficient of DIV_5*OWN10 in column (4) is positive and insignificant. In general, the results are robust to alternative variable measures.

**Table 8:** Robustness test: OWN10 as an alternative measure of ownership concentration.

	MB	MB		
	(1) Pooled	(2)SOECGs	(3)SOELGs	(4)PFs
**DIV_5**	-0.0328*** (-3.51)	-0.0991*** (-2.88)	-0.1658*** (-6.99)	-0.0058 (-0.54)
DIV_5*OWN10	0.0806*** (4.87)	0.2307*** (3.81)	0.3216*** (7.68)	0.0162 (0.86)
**OWN10**	1.6117*** (38.85)	1.6031*** (11.12)	1.3745*** (15.25)	1.4612*** (28.28)
**OWN10** ^**2**^	-1.2059*** (-26.45)	-1.3603*** (-9.10)	-1.2127*** (-13.38)	-1.0081*** (-17.37)
**TSH**	0.1741*** (13.70)	0.0736* (1.69)	-0.0249 (-0.98)	0.1920*** (12.13)
**SIZE**	-0.1258*** (-30.63)	-0.2037*** (-21.30)	-0.1290*** (-17.31)	-0.0973*** (-16.40)
**DUALITY**	0.0363*** (8.53)	-0.0593*** (-3.69)	0.0136 (1.13)	0.0366*** (7.57)
**CONTROL**	0.0080 (0.44)	-0.2818*** (-5.19)	-0.0315 (-0.76)	-0.0254 (-1.13)
**CASH**	0.0556*** (3.25)	0.1698*** (3.87)	0.0270 (0.72)	0.1293*** (5.83)
**FOREIGN**	0.0496** (2.57)	0.0520 (0.60)	0.0429 (0.81)	-0.0277 (-0.67)
**INSTITUTIONAL**	-0.0468*** (-3.64)	0.1191*** (2.68)	0.1208*** (4.49)	-0.0076 (-0.46)
**Constant**	0.1832*** (8.00)	0.6500*** (7.94)	0.3894*** (9.88)	0.1937*** (6.10)
**Year**	YES	YES	YES	YES
**Industry**	YES	YES	YES	YES
**No.of Obs.**	13527	1728	3105	8073
**Adj. R** ^**2**^	0.4318	0.4161	0.3487	0.5147

T-statistics are in the parenthesis.

*, **, *** denote significance at the 10%, 5% and 1% level respectively.

### 4.7 Controlling for the succession period of OWN1 in PFs

It is suggested that private firms may transfer control to next generation and succession tax might affect to the investment decisions, affecting firm value, so we additionally control for the succession period of ownership concentration. The results shown in Table 9 are similar to those in Table 4. The coefficient of DIV_5*OWN1 is positive but insignificant. Our results for PFs are robust.

**Table 9:** Robustness test: Controlling for the succession period of OWN1 in PFs.

MB	Coefficient	T-statistics
**DIV_5**	0.0010	0.09
DIV_5*OWN1	0.0006	0.02
**OWN1**	1.548***	22.94
**OWN1** ^**2**^	-1.707***	-19.75
**OWN1**_**i,t-1**_	-0.475*	-1.92
**TSH**	0.1506***	9.91
**SIZE**	-0.7375***	-11.55
**DUALITY**	0.0378***	6.94
**CONTROL**	-0.0599**	-2.30
**CASH**	0.2112***	8.54
**FOREIGN**	0.0156	0.34
**INSTITUTIONAL**	0.0218	1.22
**Constant**	0.3113***	8.59
**Year**	YES	
**Industry**	YES	
**No.of Obs.**	7176	
**Adj. R** ^**2**^	0.3653	

*, **, ***denote significance at the 10%, 5% and 1% level respectively.

### 4.8 Effect of stable cash dividend payout on the relation between ownership concentration and firm value

In addition to continuous cash dividends, it is suggested that stable cash dividend payout may also affect the relation between ownership concentration and firm value. We define stable cash dividend payout (STABLEDIV) as a dummy variable, which takes the value of one if dividend per share keeps the same with previous period dividend per share, zero otherwise and repeat the original MB analysis of Table 4 by replacing DIV_5 with STABLEDIV. The results are shown in Table 10. The coefficients of STABLEDIV*OWN1 in column (2), column (3) and column (4) are all positive and significant at 1% level, indicating values of SOECGs, SOELGs and PFs with stable cash dividend payout increases significantly more with ownership concentration than values of those with unstable cash dividend payout.

**Table 10:** Effect of stable cash dividend payout on the relation between ownership concentration and firm value.

	MB	MB		
	(1) Pooled	(2) SOECGs	(3) SOELGs	(4) PFs
**STABLEDIV**	-0.3124*** (-31.48)	-0.2744*** (-8.93)	-0.1937*** (-9.19)	-0.2977*** (24.25)
STABLEDIV*OWN1	0.7211*** (27.35)	0.6183*** (8.01)	0.4145*** (8.19)	0.7483*** (21.45)
**OWN1**	1.1571*** (23.78)	1.484*** (9.20)	0.7721*** (7.64)	1.2109*** (19.18)
**OWN1** ^**2**^	-1.5368*** (-26.07)	-1.989*** (9.93)	-1.1051*** (-9.76)	-1.5651*** (-19.20)
**TSH**	0.1079*** (10.02)	-0.044 (-0.13)	-0.0358* (-1.66)	0.1235*** (8.91)
**SIZE**	-0.1155*** (-27.29)	-0.1960*** (20.35)	-0.1088*** (14.21)	-0.9164*** (-15.01)
**DUALITY**	0.0368*** (8.43)	-0.0593*** (-3.67)	0.0153 (1.22)	0.0346*** (7.01)
**CONTROL**	0.0341* (8.43)	-0.2028*** (-2.77)	0.1439*** (2.66)	-0.0467** (-1.97)
**CASH**	0.1202*** (6.93)	0.1877*** (4.27)	0.0734* (1.90)	0.1903*** (8.48)
**FOREIGN**	0.0689*** (3.52)	0.0048 (0.06)	0.0443 (0.81)	-0.0021 (-0.05)
**INSTITUTIONAL**	0.0104 (0.83)	0.2294*** (5.84)	0.1492*** (5.96)	0.03840** (2.32)
**Constant**	0.4897*** (20.34)	0.8220*** (10.53)	0.5784*** (14.87)	0.4509*** (13.49)
**Year**	YES	YES	YES	YES
**Industry**	YES	YES	YES	YES
**No.of Obs.**	13527	1728	3105	8073
**Adj. R** ^**2**^	0.4058	0.4112	0.2982	0.4946

T-statistics are in the parenthesis.

*, **, *** denote significance at the 10%, 5% and 1% level respectively.

## 5 Conclusion

This paper investigates the relation between continuous cash dividends and firm value by utilizing a sample of 1503 firms listed on Chinese A-share market from 2009–2017 and examines how the relation between ownership structure and firm value is affected by continuous cash dividends with three subsamples including SOECGs, SOELGs and PFs.

This paper contains significant and robust results. First, we document a significantly positive relation between continuous cash dividends and firm value. Our results can be explained by signaling theory, free cash flow theory and agency cost theory. Signaling theory believes that cash dividend is a common and reliable signal used by firm managers to convey firms’ positive information, a useful way of gaining recognition from stock market and continuous cash dividends are more informative about a firm’s present condition and future prospects, thus increasing firm value. Free cash flow theory suggests that cash dividend can reduce free cash flows, curb overinvestment and excess consumption from firm managers and continuous cash dividends can weaken large shareholders’ ability to expropriate minority shareholders’ interest to a large extent, which is value-increasing. Agency cost theory supports that cash dividend helps alleviate the conflicts between firm managers and shareholders and continuous cash dividends can reduce agency costs so that firm value can be increased.

Second, we find continuous cash dividends have heterogeneous effects on the relation between ownership concentration and firm value based on three typical ownership types in China. Specifically, values of SOECGs and SOELGs that distribute continuous cash dividends increase significantly more with ownership concentration than values of those that distribute discontinuous cash dividends. Continuous cash dividends fail to exert any immediate effect on the relation between ownership concentration and firm value in PFs.

We also conduct several robustness tests, including endogeneity of ownership concentration and firm value, firm-fixed effects, alternative variable measures and controlling for the succession period of OWN1. We find that the results are robust.

Finally, we explore the effect of stable cash dividend payout on the relation between ownership concentration and firm value and find that stable cash dividend payout can mediate the effect of ownership concentration on values of SOECGs, SOELGs and PFs. The effect of stable cash dividend payout on the relation between ownership concentration and firm value is strongest in PFs, followed by SOECGs and lastly SOELGs.

Findings in the study provides insights into the existing literature on the effect of continuous cash dividends on firm value and the relation between ownership structure and firm value. The results suggest that firm managers in China should pay more attention to stable dividend policy for it is one way of legal protection of minority shareholders and can increase firm value and mediate the effect of ownership concentration on firm value by reducing agency costs and improving corporate governance.

## Supporting information

S1 File (RAR)
